# Physician Work Patterns in Pregnancy, Parental Leave, and Return to the Workforce

**DOI:** 10.1001/jamanetworkopen.2026.7543

**Published:** 2026-04-22

**Authors:** Andrea N. Simpson, Rinku Sutradhar, Eric McArthur, Maria C. Cusimano, Peter Tanuseputro, Nancy N. Baxter

**Affiliations:** 1 Department of Obstetrics & Gynaecology, University of Toronto, Toronto, Ontario, Canada; 2 Li Ka Shing Knowledge Institute, St Michael’s Hospital, Toronto, Ontario, Canada; 3 Department of Obstetrics & Gynaecology, St Michael’s Hospital/Unity Health Toronto, Toronto, Ontario, Canada; 4 ICES (formerly the Institute for Clinical Evaluative Sciences), Ontario, Canada; 5 Institute for Health Policy, Management and Evaluation, University of Toronto, Toronto, Ontario, Canada; 6 London Health Sciences Centre Research Institute, London Health Sciences Centre, London, Ontario, Canada; 7 Department of Surgery, Division of Gynecology, Lahey Hospital & Medical Center, Beth Israel Lahey Health, Burlington, Massachusetts; 8 Department of Family Medicine and Primary Care, University of Hong Kong, Hong Kong, China; 9 Institut du Savoir Montfort, Ottawa, Ontario, Canada; 10 Faculty of Medicine and Health, University of Sydney, Sydney, New South Wales, Australia

## Abstract

**Importance:**

Pregnancy and parental leave remain poorly supported in medical culture, yet little is known about how physicians adjust their workloads around childbirth.

**Objective:**

To examine work patterns of physicians before, during, and after pregnancy across specialties.

**Design, Setting, and Participants:**

This population-based retrospective cohort study included physicians registered with the College of Physicians and Surgeons of Ontario between April 1, 2002, and November 18, 2018, and had been practicing for at least 2 years prior to conception in Ontario, Canada. Physicians were followed up to November 26, 2023. Data were analyzed between January 2022 and December 2025.

**Exposure:**

Childbirth.

**Main Outcomes and Measures:**

Physician billing claims were used to identify the amount, cessation, and resumption of work activity around delivery. The rate of days worked per 100 person-days and rate of nights worked per 100 person-days according to physician specialty group prior to and during pregnancy were calculated. A negative binomial regression model was used to estimate the rate ratio of work activity in each trimester of pregnancy. The cumulative probability of return to the workforce according to specialty group was evaluated.

**Results:**

In this study, 5948 deliveries were evaluated among 3932 practicing physicians, median (IQR) age 35 (33-37) years at delivery, of which 2378 (40%) were first deliveries. Compared with prepregnancy, overall workload increased in pregnancy (RR, 1.06; 95% CI, 1.06-1.07). Workload increased in the first trimester (RR, 1.12; 95% CI, 1.11-1.12) and second trimester (RR, 1.12; 95% CI, 1.11-1.13) and then decreased in the third trimester (RR, 0.95; 95% CI, 0.94-0.97). Overnight work was reduced during pregnancy compared with prepregnancy (RR, 0.92; 95% CI, 0.89-0.95), although it increased in the first trimester (RR, 1.09; 95% CI, 1.06-1.12) and second trimester (RR, 1.05; 95% CI, 1.01-1.08), and decreased in the third trimester (RR, 0.62; 95% CI, 0.58-0.65). The cumulative probability of return to work was high for all specialties; surgeons returned the earliest (median [IQR], 133 [94-192] days).

**Conclusions and Relevance:**

In this cohort study, Ontario physicians appeared to frontload work in early pregnancy, likely to offset financial losses or group obligations. Wide variation in leave across specialties highlights inequities in how medical culture and practice structures accommodate childbearing. Supporting equitable, expected parental leave is critical to advancing gender equity in medicine.

## Key Points

**Question:**

How do physicians adjust their workloads before, during, and after pregnancy?

**Findings:**

In this cohort study of 5948 deliveries among 3932 practicing physicians, workload increased in the first 2 trimesters followed by a reduction in the third trimester; length of parental leave was highly variable.

**Meaning:**

These findings suggest that physicians appeared to frontload their work during pregnancy, which may reflect financial pressures or group practice obligations.

## Teaser

This cohort study examines physician work patterns before, during, and after pregnancy among physicians in Canada.

## Introduction

Pregnancy and parental leave are important health accommodations that are often poorly supported in medical culture,^1,2,3^ even though women are projected to account for half of the physician workforce in Canada by 2030,^4^ and more than half of US medical students are women.^5^ Health benefits of paid parental leave include a longer duration of breastfeeding^6^ and improved parental mental health.^7^ Although some jurisdictions support paid leave among medical trainees,^8,9^ policies and supports regarding leave and reduction in after-hours work at the end of pregnancy among practicing physicians are lacking.^10^ As a result, pregnant physicians (particularly surgeons^10^) often maintain high work intensity late in pregnancy and have short parental leaves.^11,12^ When physicians return to work, they often experience maternal discrimination,^13^ and accommodations for lactation facilities are limited.^14,15^ Physicians lose significant income while on leave, which contributes to career dissatisfaction^16^ and presents challenges to early-career physicians who often have substantial loans.^17^ The unsupportive environment may contribute to physicians leaving medicine altogether.^17,18^

A prevalent concern among physicians is placing additional burden on colleagues while taking leave in an already constrained system.^1,10^ Hence, understanding work and leave patterns is essential to physician workforce planning, as leaves of absence during a physician’s career should be expected. Our objective was to describe the pregnancy work patterns and parental leave practices of physicians in Ontario. Specifically, we sought to determine work patterns prior to and during pregnancy, as well as length of parental leave, according to specialty.

## Methods

### Study Design and Data Sources

We performed a population-based retrospective cohort study of physicians experiencing an obstetrical delivery in Ontario, Canada. The study protocol was published^19^ and approved by the Research Ethics Board at St Michael’s Hospital in Toronto, Ontario. Briefly, physicians were identified using data from the College of Physicians and Surgeons of Ontario (CPSO), the sole licensing body for all practicing Ontario physicians, and linked to the population-based databases housed at ICES (formerly the Institute for Clinical Evaluative Sciences). ICES is an independent, nonprofit research institute whose legal status under Ontario’s health information privacy law allows it to collect and analyze health care and demographic data, without consent, for health system evaluation and improvement. These datasets were linked using unique encoded identifiers and analyzed at ICES (eTable 1 in Supplement 1). This article has been written in accordance with the Reporting of studies Conducted using Observational Routinely Collected Data (RECORD) guidelines, which is an extension of the Strengthening the Reporting of Observational Studies in Epidemiology (STROBE) reporting guideline for cohort studies.^20^

### Study Population

**Figure 1.**
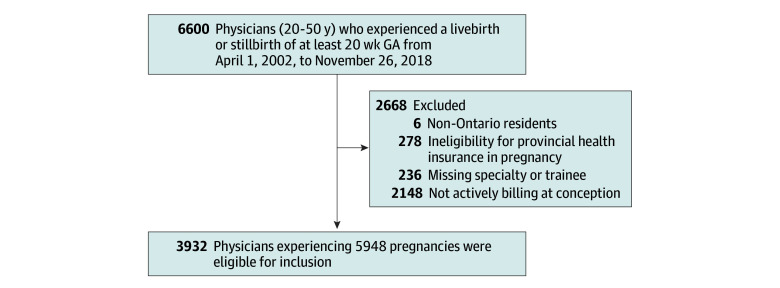
Cohort Flowchart of All Practicing Physician Deliveries in Ontario Between April 1, 2002, and November 26, 2018 GA indicates gestational age.

We included all deliveries at more than 20 weeks’ gestation among physicians in Ontario registered with the CPSO between April 1, 2002, to November 26, 2018, and followed them to a maximum follow-up date of November 26, 2023. Physicians submit billing claims to the Ontario Health Insurance Plan (OHIP), which were used to identify the amount, cessation and resumption of work activity. We restricted our cohort to physicians who had submitted claims in the 2 years prior to conception to explore work patterns prior to and during pregnancy. The date of conception was calculated by subtracting the number of gestational weeks at delivery from the delivery date. We excluded physicians with a missing specialty type. The study flowchart can be found in Figure 1.

Demographic variables were defined on the date of the index delivery. We categorized the cohort into 8 specialty groups: anesthesia or emergency medicine; diagnostic imaging; family medicine; medical specialties; obstetrics and gynecology (OB/GYN); pediatrics; psychiatry; and surgery. We evaluated maternal age, number of years in the workforce (time from first OHIP billing to conception), calendar year of delivery (divided into 2 eras: 2002 to 2009 and 2010 or later), immigration (within 5 years), comorbidities, parity (0, 1, 2 or more), multiple gestation (binary), and gestational weeks at delivery (continuous). Comorbidities were categorized into aggregated diagnosis groups (ADGs; 0, 1 to 5, 6 to 9, 10 or more) based on health care use in the 2 years prior to delivery using the Johns Hopkins ACG System Version 10.^21^

### Workload Prior to and During Pregnancy

Physician billing claims were evaluated from conception to delivery according to trimester (0 to <14 weeks, 14 to <28 weeks, ≥28 weeks, up to 294 days). We defined a referent prepregnancy period at the same calendar time of year as the pregnancy in the year prior for each individual. For example, the prepregnancy period for a physician whose date of conception was January 15, 2015, was a 294-day time period beginning on January 15, 2014. We explored workdays (any billing claim in a 24-hour period) and overnight work (claims occurring between midnight and 7:00 AM, which were identified through billing code premiums in eTable 2 in Supplement 1 and used in prior work).^22^

### Leave and Return to the Workforce

Leave was identified as the absence of billing claims following delivery. Return to work was identified through the resumption of at least 10 eligible billing claims (prefixes A, C, K, X, J) within a 1-week period.

### Statistical Analysis

We described distributions of demographic and pregnancy characteristics of physicians who experienced 1 or more deliveries according to specialty groups using medians, IQRs, and proportions. We evaluated the rate of days worked per 100 person-days in the 3 trimesters of pregnancy and prepregnancy. We used negative binomial regression with the natural logarithm of pregnancy duration as an offset in the model, and a generalized estimating equations approach under an exchangeable correlation matrix was used to account for correlation across time periods within a physician. This allowed us to estimate the rate ratio of workdays for each trimester. We repeated this process for nights worked. The median (IQR) number of nights worked during the referent prepregnancy period was also calculated for each specialty group, to aid in interpreting the associated rate ratios.

We evaluated the proportion of individuals in each specialty group that had evidence of return to work, and the median IQR of time to return to work in days. We evaluated the cumulative probability of return to work for all specialty groups. This was done under a time-to-event framework, where time zero was the date of delivery. We censored individuals if they died or lost OHIP eligibility, or if they had not returned to work within 5 years. A subsequent delivery before return to work was treated as a competing event, as this changes a physician’s risk of return to work.

We repeated this analysis for family physicians based on primary care model. In Ontario, approximately 30% of family physicians practice under models outside of fee-for-service care,^23^ which have different payment mechanisms and service requirements.^24^ There are 4 major models that may be identified using databases at ICES: (1) Family Health Network (FHN); (2) Family Health Group (FHG); (3) Family Health Team (FHT); (4) Family Health Organization (FHO). Physicians practicing under FHNs, FHTs and FHOs are paid through blended capitation, while those practicing with FHGs are paid via fee-for-service. Family physicians may also practice under other models, including pure fee-for-service, and these were captured in a fifth group (other). For all physicians, we determined the cumulative probability of return-to-work by era (2002 to 2009 and 2010 or later).

All statistical tests were 2-sided, with *P* < .05 considered statistically significant. During the cohort build, we excluded individuals with a missing specialty or physicians in training, to ensure complete case analysis. Analyses were performed using SAS 9.4 (SAS Institute). Data were analyzed from January 2022 and December 2025.

## Results

We identified 5948 deliveries among 3932 unique physicians (Table 1). The median (IQR) maternal age at delivery was 35 (33-37) years. Of the deliveries, 2378 (40.0%) were first deliveries, 2379 (40.0%) second, and 1188 (20.0%) third or higher. The median (IQR) time between entering the workforce and conception was 3.6 (2.1-5.6) years. The median (IQR) gestational weeks at delivery was 39 (38-40) weeks. Deliveries according to specialty group were as follows: anesthesia or emergency medicine (265 [4.4%]), diagnostic imaging (196 [3.3%]), family medicine (3504 [58.9%]), medical specialties (783 [13.2%]), OB/GYN (253 [4.3%]), pediatrics (382 [6.4%]), psychiatry (248 [4.2%]), and surgery (317 [5.3%]).

**Table 1.** Characteristics of Included Deliveries According to Specialty Group (N = 5948)

Characteristic	Participant, No. (%)								
	Specialty group								Overall (n = 5948)
	Anesthesia or emergency medicine (n = 265 [4.5])	Diagnostic imaging (n = 196 [3.3])	Family medicine (n = 3504 [58.9])	Medical specialty (n = 783 [13.2])	Obstetrics and gynecology (n = 253 [4.2])	Pediatrics (n = 382 [6.4])	Psychiatry (n = 248 [4.2])	Surgery (n = 317 [5.3])	
**Maternal characteristics**									
Age, median (IQR), y	36 (34-38)	37 (35-39)	34 (32-37)	36 (34-38)	36 (34-38)	35 (34-38)	36 (35-39)	37 (35-39)	35 (33-37)
Years in the workforce prior to conception, median (IQR)	2.9 (1.8-4.7)	3.7 (2.0-5.9)	3.8 (2.2-5.9)	3.4 (2.0-5.3)	3.4 (2.0-5.4)	3.1 (1.8-5.0)	3.0 (2.0-4.9)	3.7 (2.2-5.6)	3.6 (2.1-5.6)
Year of delivery									
2002-2009	73 (27.5)	82 (41.8)	1203 (34.3)	257 (32.8)	79 (31.2)	146 (38.2)	95 (38.3)	110 (34.7)	2045 (34.4)
≥2010	192 (72.5)	114 (58.2)	2301 (65.7)	526 (67.2)	174 (68.8)	236 (61.8)	153 (61.7)	207 (65.3)	3903 (65.6)
Parity^a^									
0	107 (40.4)	65 (33.2)	1441 (41.1)	314 (40.1)	96 (37.9)	124 (32.5)	105 (42.3)	126 (39.7)	2378 (40.0)
1	115 (43.4)	78 (39.8)	1354 (38.6)	350 (44.7)	97 (38.3)	158 (41.4)	109 (44.0)	118 (37.2)	2379 (40.0)
≥2	43 (16.2)	53 (27.0)	707 (20.2)	119 (15.2)	60 (23.7)	100 (26.2)	34 (13.7)	72 (22.7)	1188 (20.0)
Recent immigration (<5 y)	21 (7.9)	39 (19.9)	389 (11.1)	127 (16.2)	16 (6.3)	53 (13.9)	18 (7.3)	36 (11.4)	699 (11.8)
ADGs									
0-5	158 (59.6)	127 (64.8)	2079 (59.3)	464 (59.3)	178 (70.4)	225 (58.9)	119 (48.0)	195 (61.5)	3545 (59.6)
6-9	90 (34.0)	55 (28.1)	1217 (34.7)	277 (35.4)	69 (27.3)	138 (36.1)	111 (44.8)	104 (32.8)	2061 (34.7)
≥10	17 (6.4)	14 (7.1)	208 (5.9)	42 (5.4)	6 (2.4)	19 (5.0)	18 (7.3)	18 (5.7)	342 (5.7)
Multiple gestation (current delivery)	16 (6.0)	10 (5.1)	214 (6.1)	45 (5.7)	16 (6.3)	38 (9.9)	23 (9.3)	25 (7.9)	387 (6.5)
**Infant characteristics**									
Gestational weeks at delivery, median (IQR)	39 (38-40)	39 (38-39)	39 (38-40)	39 (38-40)	38 (37-39)	39 (38-40)	39 (38-40)	38 (38-39)	39 (38-40)

Abbreviation: ADG, aggregated diagnosis group.

^a^ Fewer than 6 physicians were missing these variables; imputed as 0 for all.

### Workload Prior to and During Pregnancy

The median number of days worked and the rate of days and nights worked per 100 person-days were calculated for each trimester of pregnancy and compared with the prepregnancy period (Table 2). Prepregnancy (14 weeks preceding the first trimester), overall workload ranged from a median (IQR) of 44 (27-57) days to 67 (35-79) days (corresponding to rates of 41.8 and 57.0 per 100 person-days). Overall workload remained stable or increased for almost all specialties in pregnancy trimesters 1 and 2 (ranging from a median [IQR] of 46 [32-57] days to 71 [55-80] days and a rate of 45.0 to 65.3 per 100 person-days) and decreased in trimester 3 (ranging from a median [IQR] of 27 [15-37] days to 43 [36-52] days and a rate of 35.9 to 58.6 per 100 person-days). Overnight work followed a similar pattern. In prepregnancy, overnight workload ranged from a median (IQR) of 0 (0-0) days to 9 (5-12) days (corresponding rates of 0.1 to 8.5 per 100 person-days). In the first 2 trimesters, overall workload ranged from a median (IQR) of 0 (0-0) days to 8 (5-11) days (rate, 0.2-8.2 per 100 person-days) in the first 2 trimesters and decreasing to a median of 0 (0-0) days to 3 (0-5) days (rate, 0.1-4.7 per 100 person-days) in the third trimester.

**Table 2.** Median Number of Workdays (Overall and Overnight) and Rate of Work Per 100 Person-Days According to Specialty for Each Trimester of Pregnancy

Specialty and pregnancy trimester^a^	All workdays		Overnight work	
	No. of workdays, median (IQR)	Rate per 100 person-days	No. of workdays, median (IQR)	Rate per 100 person-days
Anesthesia or emergency medicine				
Prepregnancy	46 (36-55)	43.6	4 (2-9)	6.7
First	46 (39-53)	47.1	5 (2-9)	6.9
Second	47 (39-54)	47.3	4 (2-9)	6.3
Third	27 (19-36)	36.5	1 (0-3)	3.2
Diagnostic imaging				
Prepregnancy	67 (35-79)	57.0	0 (0-1)	1.1
First	69 (54-79)	62.0	0 (0-1)	1.3
Second	71 (55-80)	62.8	0 (0-1)	1.4
Third	46 (28-58)	56.6	0 (0-0)	1.1
Family medicine				
Prepregnancy	54 (37-66)	50.0	0 (0-3)	3.1
First	55 (41-65)	52.5	0 (0-3)	3.2
Second	55 (41-66)	52.6	0 (0-2)	3.1
Third	35 (21-47)	45.3	0 (0-1)	1.9
Medical specialty				
Prepregnancy	53 (35-67)	50.7	0 (0-1)	1.7
First	55 (38-68)	53.3	0 (0-1)	1.6
Second	55 (39-68)	53.8	0 (0-1)	1.5
Third	36 (22-47)	46.3	0 (0-0)	1.0
Pediatrics				
Prepregnancy	44 (27-57)	41.8	0 (0-4)	2.8
First	46 (32-57)	45.0	0 (0-4)	2.9
Second	47 (32-57)	45.3	0 (0-3)	2.8
Third	27 (15-37)	36.1	0 (0-1)	1.4
Psychiatry				
Prepregnancy	48 (29-58)	42.2	0 (0-0)	0.1
First	48 (34-57)	44.6	0 (0-0)	0.2
Second	47 (33-58)	45.0	0 (0-0)	0.2
Third	27 (16-38)	35.9	0 (0-0)	0.1
OB/GYN				
Prepregnancy	66 (52-74)	60.6	9 (5-12)	8.5
First	67 (57-74)	64.1	8 (5-11)	8.2
Second	67 (58-75)	65.3	8 (4-11)	7.9
Third	43 (36-52)	58.6	3 (0-5)	4.7
Surgery				
Prepregnancy	62 (48-70)	58.0	1 (0-4)	2.6
First	63 (50-70)	59.6	1 (0-3)	2.2
Second	62 (51-70)	60.3	1 (0-3)	2.3
Third	39 (27-49)	51.5	0 (0-1)	1.4

Abbreviation: OB/GYN, obstetrics and gynecology.

^a^ Trimesters as follows: Conception to 13 weeks and 6 days gestation, 14 to 27 weeks and 6 days gestation, 28 weeks gestation until delivery. Prepregnancy time period is the 14 weeks leading up to the first trimester.

**Figure 2.**
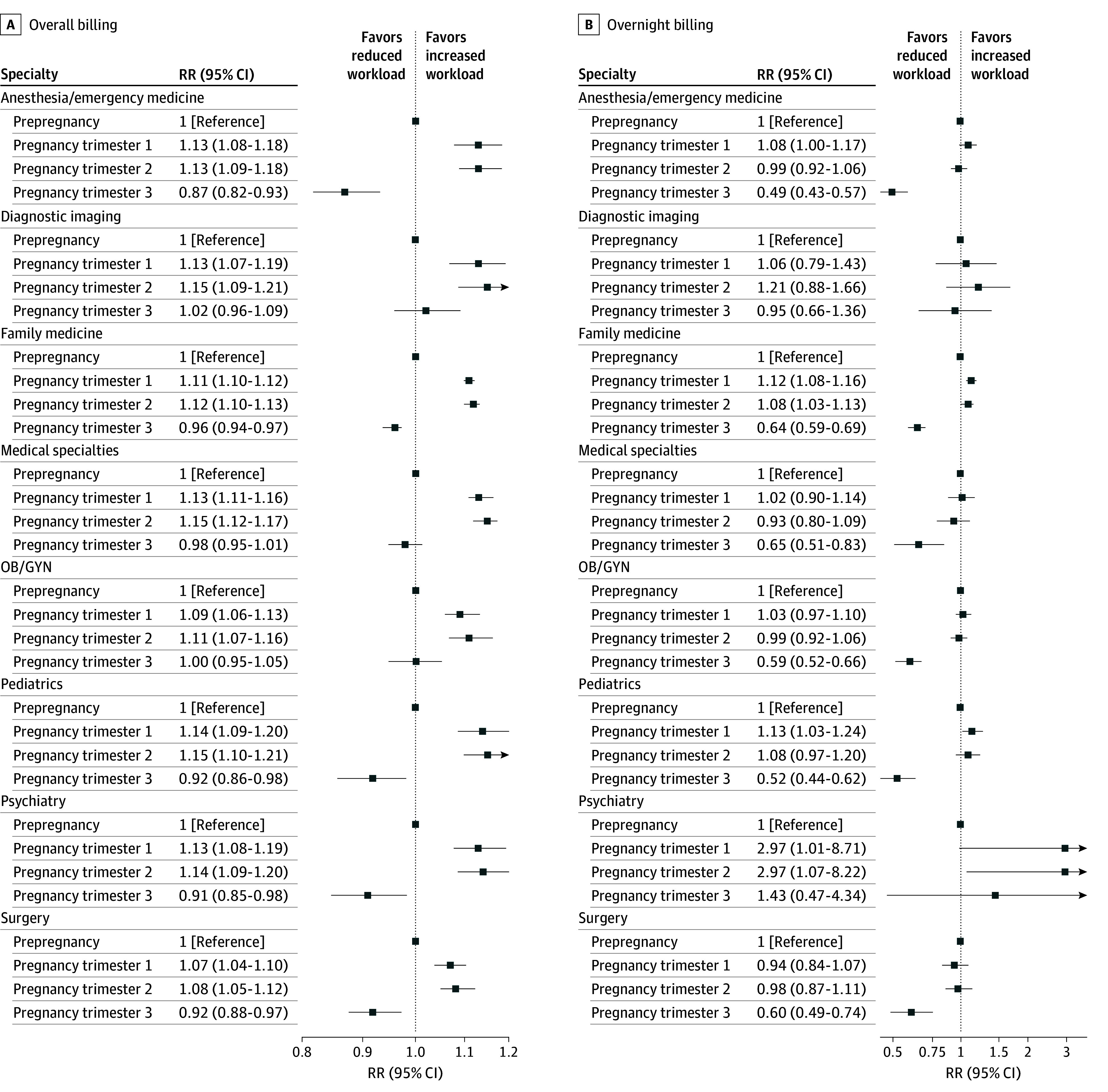
Forest Plot Demonstrating the Rate Ratio (RR) of All Workdays and Overnight Work Prior to and During Pregnancy According to Specialty Group OB/GYN indicates obstetrics and gynecology.

The rate ratios of overall and overnight work per trimester and prepregnancy are presented in Figure 2. Among all physicians, overall workload increased in pregnancy (RR, 1.06; 95% CI, 1.06-1.07) compared with prepregnancy. When examined across trimesters, workload increased in the first (RR, 1.12; 95% CI, 1.11-1.12) and second (RR, 1.12; 95% CI, 1.11-1.13) trimesters and then decreased in the third trimester (RR, 0.95; 95% CI, 0.94-0.97) compared with prepregnancy. This pattern was seen in all specialty groups except for diagnostic imaging (RR, 1.02; 95% CI, 0.96-1.09; *P* = .48), medical specialties (RR, 0.98; 95% CI, 0.95-1.01; *P* = .20), and OB/GYN (RR, 1.00; 95% CI, 0.95-1.05; *P* = .99), where the rate ratios in the third trimester returned to the prepregnancy referent baseline.

Compared with prepregnancy, overnight billings were reduced in pregnancy overall (RR, 0.92; 95% CI, 0.89-0.95). Similar to the pattern seen for overall workload, overnight work rate ratios remained stable in pregnancy trimesters 1 (RR, 1.09; 95% CI, 1.06-1.12) and 2 (RR, 1.05; 95% CI, 1.01-1.08), and decreased in pregnancy trimester 3 (RR, 0.62; 95% CI, 0.58-0.65); this was true for all specialties except psychiatry and diagnostic radiology. For psychiatry, the rate ratio increased compared with prepregnancy in all 3 trimesters (RR, 2.97 [95% CI, 1.01-8.71]; RR, 2.97 [95% CI, 1.07-8.22]; and RR, 1.43 [95% CI, 0.47-4.34], respectively); however, the rate of overnight work per 100 person-days was lowest for psychiatry in all time periods at 0.2 in the first 2 trimesters and 0.1 in the third trimester. For diagnostic imaging, overnight billing remained stable throughout pregnancy. The overnight work rate ratio in pregnancy trimester 3 was lowest for anesthesia or emergency medicine (RR, 0.49; 95% CI, 0.43-0.57; *P* < .001), with the rate per 100-person days dropping from 6.9 per 100-person days in trimester 1 to 3.2 per 100-person days in trimester 3.

### Leave and Return to the Workforce

**Figure 3.**
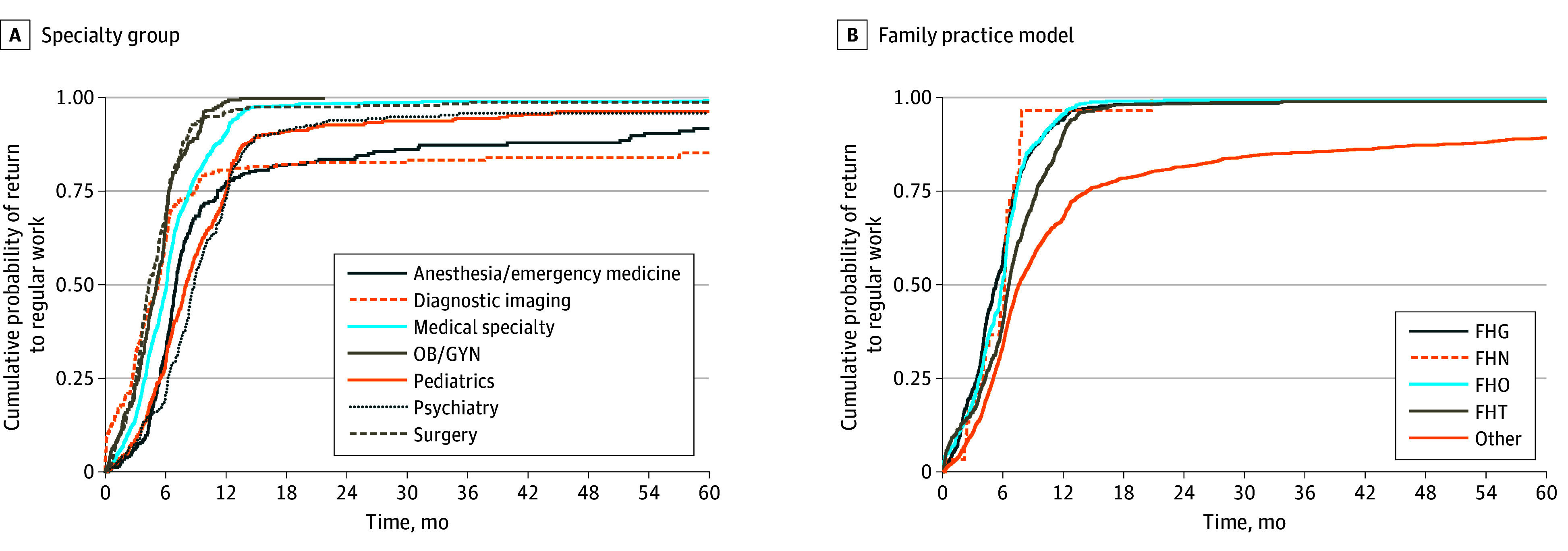
Line Graph of Cumulative Probability of Return to the Workforce According to Specialty Group and Family Practice Model FHG indicates Family Health Group; FHN, Family Health Network; FHO, Family Health Organization; FHT, Family Health Team; OB/GYN, obstetrics and gynecology.

Physicians across all specialties had high proportions of return to work during the follow-up period (Figure 3A). The median (IQR) number of days to return to the workforce ranked lowest to highest were: surgery, 133 (94-192) days; OB/GYN, 156 (101-193) days; diagnostic imaging, 161 (84-276) days; medical specialties, 187 (122-258) days; family medicine, 199 (128-312) days; anesthesia or emergency medicine, 214 (167-340) days; pediatrics, 245 (168-366) days; and psychiatry, 270 (191-372) days.

Within family medicine (Figure 3B), most family physicians practicing within primary care models returned to work. Family physicians practicing in other models had a lower proportion return to the workforce during the follow-up period (Figure 3B). The median (IQR) number of days ranged from 160 (103-216) days for FHG, 179 (110-222) days for FHO, 186.5 (121-214) days for FHN, and 199 (128-283) days for FHT and were longest in family physicians practicing outside of these models at 228 (152-430) days.

The cumulative probability of return to work by 180 days postpartum was 47.2% (965 of 2045) among physicians who delivered between 2002 and 2009 compared with 39.6% (1545 of 3903) among physicians who delivered in 2010 or later. By 365 days, return to work was similar for both eras (1717 of 2045 [84.0%] and 3192 of 3903 [81.8%], respectively) (eFigure in Supplement 1).

## Discussion

Patterns of physician work in pregnancy and parental leave practices have not been well-studied, particularly among practicing physicians, and our work has implications for physician workforce planning. We observed that physicians in all specialties increased workload during trimesters 1 and 2 compared with prepregnancy. Almost all specialty groups decreased workload in trimester 3, except for diagnostic imaging, medical specialties, and OB/GYN. Overnight work appeared to remain stable for almost all specialties in the first 2 trimesters, followed by a decrease in the third trimester. Surgeons, OB/GYNs, and radiologists returned to the workforce soonest following delivery. Within family medicine, physicians practicing within major primary care models exhibited high rates of return to work compared with physicians outside of these models. Parental leaves were slightly longer in more recent years (among physicians delivering in 2010 or later compared with those delivering between 2002 and 2009).

In this study, approximately half of the deliveries were first deliveries, with the median (IQR) age of 35 (33-37) years and 3.6 (2.1-5.6) years in the workforce at the time of delivery. This is congruent with prior research, as physicians often delay childbearing to accommodate their years of training.^25^ Hence, many physicians will experience more than 1 parental leave during practice, and negotiations on a government level to plan for the number of physicians needed to sustain patient care should account for these leaves. With extended training pathways overlapping with the optimal age of reproduction, physicians-in-training should be encouraged to consider family planning at an earlier point in their careers,^1^ and improving medical culture to accommodate physicians who wish to become parents at any career stage should be prioritized. In academic medicine, improved parental leave support may contribute to physician retention and likelihood of promotion.^26^

Currently in Ontario, there are limited financial supports available to practicing physicians. Standard parental leave for birthing parents in Ontario is 12 to 18 months, with parental leave pay provided through federal employment insurance; while this applies to resident physicians, it does not apply to most practicing physicians in Ontario. A limited stipend is provided to practicing physicians in Ontario, which was $1000 per week for a maximum of 17 weeks prior to 2025. The observed increase in workload in the first 2 trimesters of pregnancy suggests that physicians prepare for loss of income and ability to work as intensely in the third trimester. Increased workloads early in pregnancy could also reflect a sense of debt to one’s colleagues prior to a planned leave. In surgical culture specifically, prioritization of patients and colleagues over one’s own personal needs is a common experience.^27^ Similar to prior literature, surgeons and OB/GYNs are among the first groups to return from parental leave, with a median time of 4.5 to 5.5 months. The majority of physicians, regardless of specialty, returned to work within 12 months of childbirth. By comparison, less than 10% of Canadians receiving parental leave pay return to the workforce before 8 months, with the majority returning between 9 to 12 months or later.^28^ Paid leave has been identified as a strategy to attract and retain employees in industries outside of medicine.^29^ Many large tech companies have introduced gender-specific health benefits, such as insurance for fertility services and oocyte cryopreservation,^30^ to encourage the retention and promotion of women employees through increased reproductive autonomy. Encouragement of paternity leave has also been identified as a method to improve gender equity in the workforce.^31^ Similar policies and benefits would be highly applicable to physician leave as well. For physicians, proposed changes may include the creation of standard policies to promote adequate length of leave (including paternity leave),^11,16,27^ increasing physician compensation during leave,^11^ and measures to increase flexibility in workload for physicians when a group member is on leave.^17^ Flexibility on a population level could be increased through increasing the number of available physicians, group practice models where appropriate, and funding for temporary replacements to ease burden on groups when a colleague is on leave.

### Limitations

This study has limitations, which include an inability to determine parental leave patterns among resident physicians, as they do not submit billing claims. We could not determine parental leave practices among male physicians, but this is an important consideration in physician workforce planning, as recent data have suggested that married male physicians also adjust their workload to accommodate parenting.^32^ We were unable to evaluate leave practices for physicians who became parents through surrogacy or adoption. We were unable to determine whether any local policies within groups of physicians influenced leave patterns. It is notable that family physicians practicing within a major primary care model appeared to have higher rates of return to work. Further work to understand practice models for all specialties that are more conducive to supporting physician leaves is needed to foster a more supportive culture. We did not assess whether the COVID-19 pandemic influenced length of parental leaves among physicians, and therefore this work reflects parental leave patterns outside of pandemic times. Finally, our work is restricted to Ontario physicians and may not be generalizable to other practice settings.

## Conclusions

In this cohort study of Ontario physicians, we found that physician workload increased in the first 2 trimesters of pregnancy, which may reflect financial pressures or obligations within physician groups to frontload work prior to leave. Parental leave practices were highly variable among physicians in Ontario and significantly shorter than leaves for the average Canadian receiving parental leave pay; this is likely a reflection of the culture of medicine and the constrained medical system. These issues are likely even more prevalent in other jurisdictions, such as the US, where adequate paid parental leave options for physicians are even more limited.^33^ Ensuring adequate parental leave for physicians is a gender equity issue and should be addressed through workforce planning at a population level to ensure adequate physician supply, improved financial support for physicians on leave, structuring practice models in a way that supports leave for physician parents, as well as through the standardization of policies through professional societies and advocacy for recommended leave practices.

## Notes

Supplement 1. **eTable 1. **Databases Used in This Study **eTable 2.** Codes to Identify After-Hours Work in the Ontario Health Insurance Plan (OHIP) Schedule of Benefits **eFigure. **Cumulative Probability of Return to Workforce by Era (2002-2009 vs ≥2010)

Supplement 2.
Data Sharing Statement
