# Supplementary material for: Physician Work Patterns in Pregnancy, Parental Leave, and Return to the Workforce
Source: JAMA Netw Open. 2026 Apr 22;9(4):e267543. doi: 10.1001/jamanetworkopen.2026.7543 (PMC13103817; doi:10.1001/jamanetworkopen.2026.7543)
Supplement: Supplement 1. — eTable 1. Databases Used in This Study eTable 2. Codes to Identify After-Hours Work in the Ontario Health Insurance Plan (OHIP) Schedule of Benefits eFigure. Cumulative Probability of Return to Workforce by Era (2002-2009 vs ≥2010) [file jamanetwopen-e267543-s001.pdf]

Supplemental Online Content

Simpson AN, Sutradhar R, McArthur E, Cusimano MC, Tanuseputro P, Baxter NN. Physician work patterns in pregnancy, parental leave, and return to the workforce. *JAMA Netw Open*. 2026;9(4):e267543. doi:10.1001/jamanetworkopen.2026.7543

eTable 1. Databases Used in This Study

eTable 2. Codes to Identify After-Hours Work in the Ontario Health Insurance Plan (OHIP) Schedule of Benefits

eFigure. Cumulative Probability of Return to Workforce by Era (2002-2009 vs ≥2010)

This supplemental material has been provided by the authors to give readers additional information about their work.

© 2026 Simpson AN et al. *JAMA Network Open*.

eTable 1. Databases Used in This Study

Database	Source	Variables
College of Physicians and Surgeons of Ontario (CPSO)-all licensed physicians from January 1, 1990 to November 26, 2018	CPSO	Physician occupation<br>Specialty
MOMBABY	ICES - derived from the Canadian Institutes for Health Information Discharge Abstract Database (CIHI DAD)	Obstetrical deliveries
Registered Persons Database (RPDB)	ICES	Demographic information
CENSUS	ICES	Postal code of residence
Immigration, Refugees and Citizenship Canada (IRCC) Permanent Resident Database	ICES	Immigration status
Ontario Health Insurance Plan (OHIP)	ICES	Physician billings<br>Overnight/ after-hours work<br>Comorbidities
CIHI DAD	ICES	Prior healthcare utilization and comorbidities
National Ambulatory Care Reporting System (NACRS)	ICES	Comorbidities

© 2026 Simpson AN et al. *JAMA Network Open*.

eTable 2. Codes to Identify After-Hours Work in the Ontario Health Insurance Plan (OHIP) Schedule of Benefits

**Covariate/Outcome**	**OHIP**
Overnight work<br><br>*One night of overnight work defined as a night in which at least 1 relevant OHIP code was billed	Physician Office: A964, A996<br><br>Home Visit: B964, B996<br><br>Palliative Care Home Visit: B966, B997<br><br>Geriatric Home Visit: B986, B987<br><br>Diagnostic Services: C104, C107, C110<br><br>Hospital Inpatient: C964, C996, C997<br><br>Surgical Assistant: E401, C999<br><br>Anaesthesia: E401, C999<br><br>Other Premiums: E413, E410, E403, E408, G556<br><br>Family Practice: H122, H123, H121, H124, H122<br><br>ER by ER: H964, H986, H987<br><br>ER by MD: K964, K996, K997<br><br>Other Setting: Q964, Q996,<br><br>Hospital Outpatient: U964, U996, U997<br><br>Long-Term Care Institution: W964, W996, W997

© 2026 Simpson AN et al. *JAMA Network Open*.

eFigure. Cumulative Probability of Return to Workforce by Era (2002-2009 vs ≥2010)

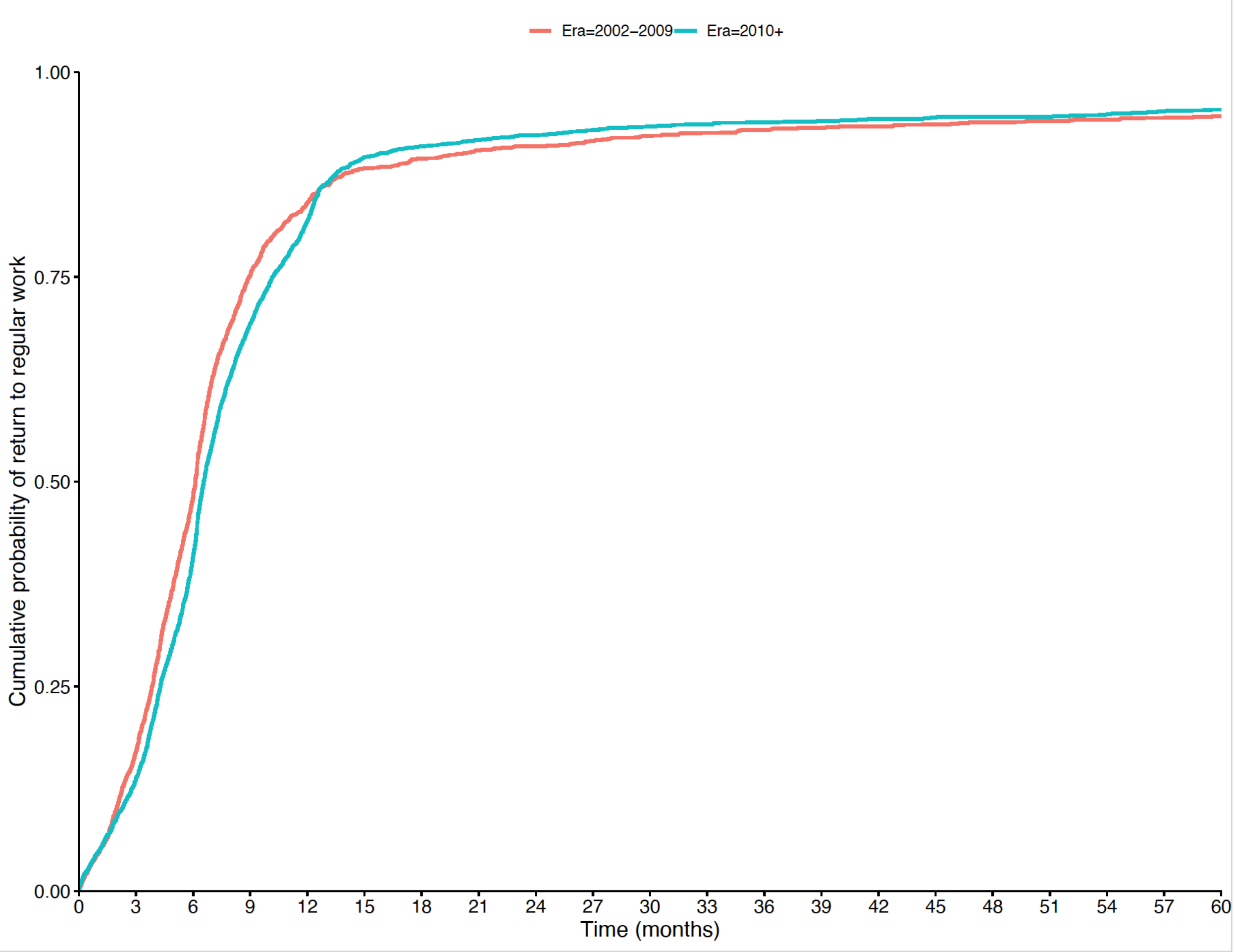

Return to work was identified through the resumption of at least 10 eligible billing claims within a 1-week period.

© 2026 Simpson AN et al. *JAMA Network Open*.
